# Access to palliative care in patients with advanced cancer of the uterine cervix in the low- and middle-income countries: a systematic review

**DOI:** 10.1186/s12904-023-01263-9

**Published:** 2023-09-20

**Authors:** Francis Ooko, Tebogo Mothiba, Peter Van Bogaert, Johan Wens

**Affiliations:** 1 Faculty of Health Sciences, University of Limpopo, Private Bag X1106, Sovenga, 0727 South Africa; 2 Faculty of Medicine and Health Sciences, University of Antwerp, Prinsstraat 13, Antwerp, 2000 Belgium

**Keywords:** Cervical cancer, Access, Palliative care, Low and middle-income countries

## Abstract

**Background:**

Women with advanced uterine cervical cancer suffer from a combination of moderate to severe physical, psychological, social, and spiritual distress due to their disease and are in need of palliative care to improve their quality of life. Approximately 85% of the women live in the low- and middle-income countries. Whether these women and their families access palliative care is not known.

**Objectives:**

To understand the geographic accessibility, availability, financial accessibility, and acceptability of palliative care by patients with advanced cervical cancer and their families.

**Methods:**

We conducted a Systematic review following PRISMA guidelines in CINAHL, Cochrane Central Register of Controlled Trials, MEDLINE, PsychINFO, PubMed and Scopus for the core concepts: palliative care, access, advanced uterine cervical cancer. Eligible articles were published in English, contained original data on experiences of patients and/or caregivers including symptoms management, and discussed available resources, communication, satisfaction, and healthcare utilization.

**Results:**

Overall there was limited access to palliative care with the few available facilities located in cities, far from the rural areas where most women lived. Pervasive poverty was common with poor affordability of healthcare, travelling, accommodation, and subsistence expenses. Misconceptions and poor knowledge of the disease, cultural beliefs and attitudes, and other health system insufficiencies also presented challenges for access.

**Conclusion:**

Concerted effort should be made to improve availability of palliative care facilities. Health education to address misconceptions and other cognitive barriers that limit access among cervical cancer patients and their families should be urgently undertaken in the LMICs.

**Supplementary Information:**

The online version contains supplementary material available at 10.1186/s12904-023-01263-9.

## Introduction and background

Cervical cancer is the second most prevalent cancer in women in South Africa. Recent data reveal that 6945 cases of cervical cancer were diagnosed in 2019 being 15.85% of all histologically diagnosed cancers (Cancer Statistics NICD, 2022: https://www.nicd.ac.za), with an estimated mortality of 19.6 per 100,000 population (Global Cancer Observatory, 2022: https://gco.iarc.fr). Therefore, cervical cancer is a public health problem in South Africa.

Although cervical cancer is a preventable disease, it still causes significant morbidity and mortality in women, particularly in the low- and middle-income countries (LMICs) where up to 88% of all global cervical cancer related deaths occur [1, 2]. High prevalence of symptoms such as vaginal discharge, vaginal bleeding, and spiritual distress are found in women living with or who die from cervical cancer [2]. Clinically evident anxiety, depressed mood, sexual dysfunction, and abandonment are common among these patients [3] in addition to financial distress particularly among patients and their families living in less developed countries [4]. Globally, approximately 2.6 million patients living with and 250 000 who died from cervical cancer in 2017 reported a combination of moderate to severe physical, psychological, social, and spiritual distress of whom 85% were in the LMICs [5]. Nevertheless, many other studies worldwide report significant unmet physical, psychosocial, and spiritual needs among cervical patients and their families [6–8]. Such report exemplify the need to provide palliative care (PC) alongside specific cancer therapy in patients with advanced cancer of the uterine cervix to alleviate distress and achieve for them good quality of life (QoL). Therefore, access to PC according to an individual’s need is critical in patients with cervical cancer and their families throughout the disease trajectory.

Palliative care is “the active holistic care of individuals across all ages with serious health-related suffering due to severe illness and especially of those near the end of life. It aims to improve the quality of life of patients, their families, and their caregivers” [9]. Earlier studies provide evidence that provision of PC alongside usual oncologic care significantly improves QoL of patients and their families [10–12]. However, access to PC is not universally guaranteed for all in need due to complex factors such as poor referral system, lack of physical facilities, poor general public awareness, negative beliefs, and other sociocultural factors [13–16]. Furthermore, lack of PC knowledge and skills have been reported among a large number of healthcare professionals in many LMICs [17]. Other factors known to affect access include geographic distance from health facilities, lack of medicine, poor affordability of services, lack of information on the specific disease, and poor healthcare professionals’ attitude, knowledge and skills [18].

**Fig. 1:**
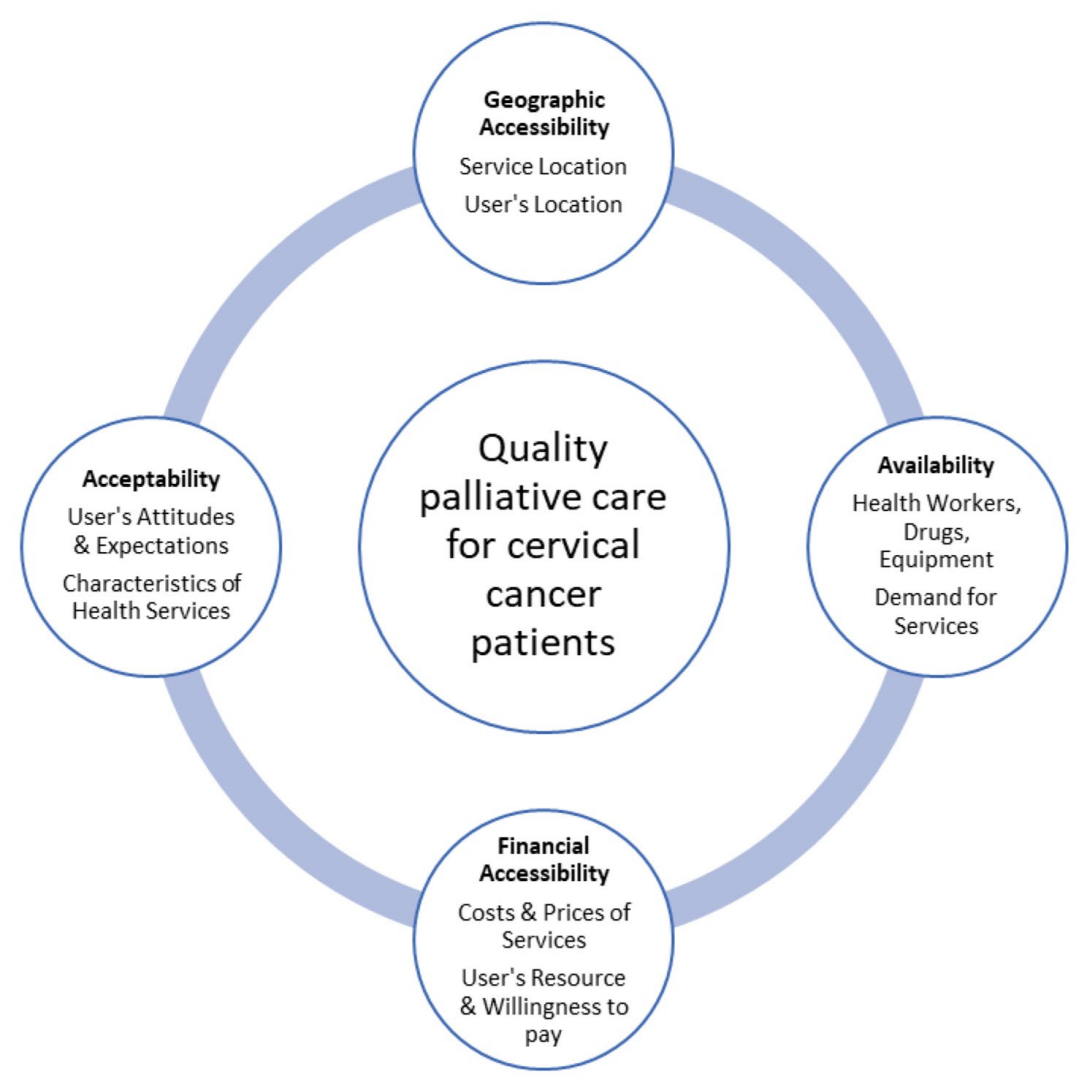
Conceptual model for access to quality healthcare services in developing countries. Adapted with permission from Peters et al. [24]

Disparities in access to PC by women with cervical cancer from different resource settings have been studied [18, 19]. We conducted this systematic review to gain a better understanding of barriers, facilitators, and measurable factors that can act as basis of remedial actions for improving access to PC by cervical cancer patients and their families living in the LMICs. In particular, this review aims to identify the physical availability of PC facilities and the enablers and barriers to utilization of these services guided by the conceptual model of access to quality healthcare in developing countries (Fig. 1).

The review question, PICOTS, and inclusion/exclusion criteria are presented in Table 1.

**Table 1:** Study eligibility criteria

**Review question**: What factors are important for access to palliative care by women with advanced cancer of the uterine cervix in the low- and middle-income countries?
**Population**: Women diagnosed with advanced cancer of the uterine cervix (advanced cancer being defined as any of the following: FIGO stage IB to IV; or presence of distant metastasis; or cancer that is life- limiting; or cancer with prognosis of 6 to 12 months); Age 19 years and older (this age group defines and adult); and/or family or caregiver of the patient; patient and caregiver residing in the LMICs. LMICS are classified as countries with Gross National Income per capita of US$1135 or less to US$13845 (https://datahelpdesk.worldbank.org/knowledgebase/articles/906519-world-bank-country-and-lending-groups). Caregiver is defined as family or any person who attended to the needs of the patient during her illness **Intervention(s), exposure (s)**:Any care given to relieve distressing symptoms, – physical or otherwise, before, alongside, or after the disease specific treatment, such as pain management, vaginal bleeding, vaginal discharge, and psychosocial support **Comparator**: Palliative care in any form is not provided **Outcome**: Specified or unspecified aspects of palliative care outcomes and/or associated outcomes such as problems or facilitators of access; physical or psychosocial needs; cultural factors; communication of needs – with family, healthcare staff, or other patients; underserved/minority groups – ethnic or socio-economic factors **Outcome measures**: Qualitative and/or quantitative with regard to availability, affordability, physical accessibility and acceptability of the care; primary palliative care use - access, provision, implementation, integration **Setting**: Home, hospice facility, outpatient oncology setting, outpatient non-oncology setting, in-patient oncology setting, and inpatient non-oncology setting
**Inclusion criteria**: • Original research involving data on adult women with advanced stage uterine cervical cancer, and/or their caregivers • Studies of cancer patients, or gynecological cancer patients with an arm including patients with cervical cancer • Studies conducted on patients and/or caregivers living in the LMICs. • Any care intended to relieve distressing symptoms • Outcome includes qualitative or quantitative measures • Published anytime from inception of database • Study design can include experimental studies (randomized controlled trials, other types of trials), observational studies (cohort studies, case-control studies, controlled and uncontrolled pre/post studies), retrospective studies, cross-sectional studies, qualitative, quantitative, mixed-method studies
**Exclusion criteria** : • Trials of medications or medical procedures related to primary cancer therapy • Case reports, editorials, letters to the editor, commentaries, historical perspectives, reviews, gray literature including guidelines and protocols, newspaper articles, social media • Review articles (not systematic review) • Diseases other than cancer of cervix • Studies reporting factors not expressed by patients or caregivers • Papers published in any language other than English

## Methods

The study proposal is registered in the International Prospective Register of Systematic Reviews (https://www.crd.york.ac.uk/prospero/display_record.php?ID=CRD42022310163).

### Search strategy

**Fig. 2:**
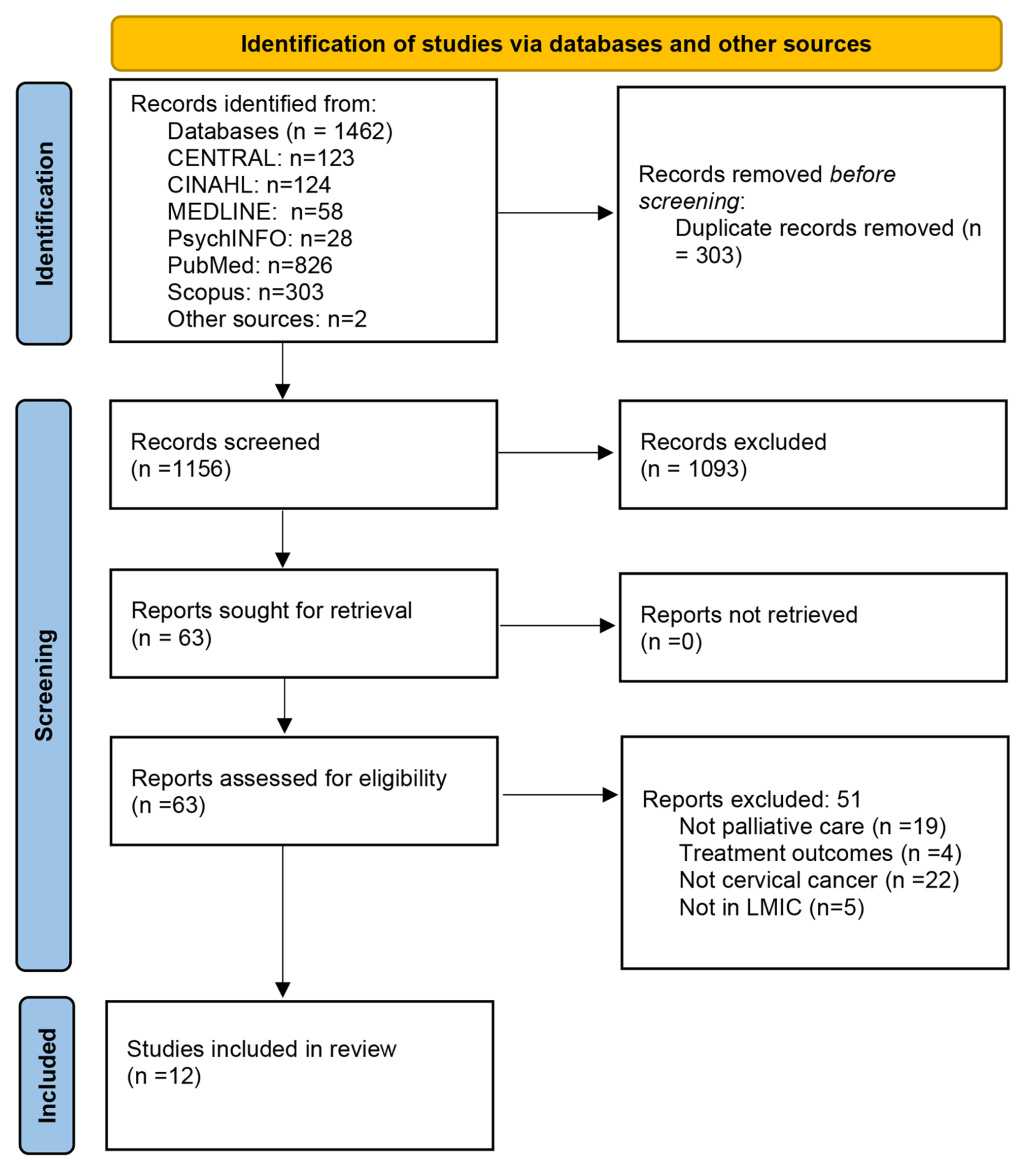
PRISMA Flow diagram. Adapted from Page et al. [20]

The updated Preferred Reporting Items for Systematic Reviews and Meta-analysis (PRISMA) 2020 statement guideline for systematic reviews [20] was followed to standardize the search and reporting process. Literature search was conducted in six electronic databases (Cumulative Index of Nursing and Allied Literature [CINAHL], Cochrane Central Register of Controlled Trials [CENTRAL], MEDLINE, PubMed, PsychINFO and Scopus) in May 2022. Advanced Boolean search strategy with at least the following terms using controlled vocabulary and free text was used: (((woman OR women OR female*) AND (neoplasm* OR oncology OR cancer OR tumo?r or malignanc*)) AND (palliative care OR end of life care OR terminal care OR hospice care OR palliati*) AND (cervi*)). Second line of search among records after screening of titles and abstract done using search terms: access* OR availability OR available OR barriers OR utilization OR utilize*. Additional hand searches of the reference lists of the included studies was also done to identify relevant articles not initially picked up by electronic search. Overview of the search strategy is shown in Fig. 2.

### Study selection

Search results were uploaded onto EndNote™20 to identify and remove duplicates, and perform further screening. The remaining articles after deduplication were screened for eligibility based on the criteria in Table 1. Two authors (F.O. and P.B) screened the titles, abstracts, and full-text articles for eligibility, and reached a consensus on the included articles.

### Quality appraisal

Due to heterogeneity of the selected articles, different methods were used to assess the methodological quality of the included studies. The Strengthening Reporting of Observational Studies in Epidemiology (STROBE) statement [21] was used for observational studies, whereas Standards for Reporting Qualitative Research (SRQR) checklist [22] was used for qualitative studies. Studies that scored at least 75% in the respective checklists were considered of good methodological quality and included in the final analysis. Analysis of quality appraisal are demonstrated in the supplementary files.

### Data extraction

Selected articles were heterogeneous with both quantitative and qualitative designs thus not allowing either meta-analysis of the quantitative studies or meta-synthesis of the qualitative studies. A textual narrative approach was used to synthesize the different types of research evidence for qualitative, quantitative, and mixed-method studies [23]. For each included article, the first author (F.O) extracted data into a preformatted table. Data fields included information of authors, publication year, country, study aims, design, participants, themes, and study findings. Findings were sorted and grouped under themes describing domains of access to healthcare in developing countries [24][Fig. 1]. The third author (P.B) checked the results and discussed any changes and revisions until consensus was reached.

## Results

Results of the study selection process are reported according to the PRISMA statement updated guidelines [20]. Excluding duplicates, database searches produced 1156 records (Fig. 2). Following title and abstract screening 63 articles remained and were downloaded for full-text screening, out of which 12 articles met final inclusion criteria and were analysed. Three of these articles [18, 25, 26] emanated from one large study with each article reporting on participant groups investigated with different experimental designs. Study characteristics and findings are summarized in Table 2.

**Table 2:** Summary of study design, aims and objectives, participants, themes and findings

Author, Year, Country	Aims	Design	Participants	Themes	Findings
Kabebew et al. [29], 2022, Ethiopia	To evaluate the satisfaction of family caregivers of patients with advanced cancer of the cervix in a tertiary hospital	Prospective cross-sectional	385 patients with stage IIB –IVB cervical cancer and 360 primary caregivers	Limited support from intimate partners Financial constraints Advanced disease Availability of care Poor communication and physical symptom care Long interval to onset of treatment Positive attitude of healthcare providers	Spouses constituted 13.6% 0f caregivers Most caregivers (66.7%) work for upkeep Most patients (75%) have stage III and IV cancers High satisfaction with psychosocial care and availability of care Lower satisfaction with information provision and physical care Least satisfaction with time taken to reach diagnosis Most caregivers satisfied with availability of nurses to the care givers and doctors to the patients
Bates et al. [4], 2021, Malawi	Investigate whether total household cancer-related health care costs over 6 months after a diagnosis of advanced cancer are associated with a patient receiving palliative care, and the experience of catastrophic costs in the household	Prospective cross-sectional study	150 households with 150 patients with advanced cancer, and 121 family caregivers. At 6 months 89 households with 89 patients and 64 caregivers were evaluable, out of which 60 patients (67%) had cervical cancer	Socially active people with responsibility People live in rural areas Limited facilities for cancer care countrywide Access available but limited Limited support. Patients depend on own resources for some needs People in urban areas within 50 km radius more likely to access services Majority of households living in extreme poverty Most patients/households experienced financial hardship More non-PC recipient households used a larger proportion of annual income on healthcare Fewer households receiving PC experienced catastrophic costs Some households chose to forgo treatment rather than have catastrophic costs More rural households associated with catastrophic costs Non-PC recipient households associated with dissaving	Median patient age was 50 years (IQR 40–57); 47 (73%) of caregivers were female 48 households were rural Cancer care and PC facilities only available in the 4 tertiary hospitals Free treatment offered supported by Government and charitable groups Limited additional needs supported by charities 9 households received PC while 70 did not 89 households lived in extreme poverty (≤ $1.89 per day) Median annual household income before illness: • Overall - $204 (IQR 84–660) • PC recipients - $537 (07-821) • Non-PC recipients- $179(82–537) Household healthcare costs after 6 months: • PC patients - $50 (11–101) Direct costs $6 (4–26) Indirect costs $36 (5–56) • Non-PC patients - $55 (28–91) Direct costs $12 (0–21) Indirect costs $33 (13–56) Total healthcare cost as a proportion of total income • PC patients − 0.086 (0.037–0.579) • Non PC-patients − 0.278 (0.085–0.692), p = 0.126 Households experiencing catastrophic costs • PC – 9 of 19 (47%) Non-PC − 48 of 70 (69%), p = 0.109 • Rural – 37 of 48 (77%) Urban – 20 of 41 (49%), p = 0.008 Median dissaving at 6 months per household • PC - $11 (0–36) Non-PC - $34 (14–75), p = 0.005
Tapera et al. [25], 2021 Zimbabwe	To investigate some key and contextual strategies which could be implemented to improve access and uptake of cervical cancer treatment and palliative care by women	Qualitative inquiry as part of a sequential explanatory mixed method study	84 purposively enrolled participants: 16 in-depth interviews with cervical cancer patients/caregivers and 20 key informants (health workers, policy makers and spiritual leaders 6 focus group discussions of cervical cancer patients, caregivers and male partners	Poor affordability Out-of-pocket expenditure on healthcare costs Poor access to definitive care Treatment unavailable to people in rural areas High costs of transport Accommodation costs Loss of income for patients and family members Limited knowledge and awareness by healthcare workers (HCW) Alternative care due to financial hardship Limited information on disease Limited resources Equipment unavailable Skills deficiency; no team approach to patient care Treatment guidelines and guidance unavailable Unmet needs	Treatment associated with high cost which most patients could not afford Most patients relied on out of pocket funding for their treatment Most patients could not afford cost of histopathology tests to confirm cancer before treatment decision is made Servicers are centralized in major cities out of reach of most rural people Cost of transport to reach centralized services unaffordable Cost of accommodation while attending services in cities especially for out of town rural people Patients and accompanying family members unable to earn income Healthcare professionals not well informed about cervical cancer Seeking alternative medicine (traditional and spiritual healers) as people cannot afford conventional treatment for cervical cancer Patients and families are not given adequate information about their illness and treatment available to them Few cancer treatment and palliative care centres Frequent equipment breakdown at the few facilities Different disciplines required for PC such as social workers and psychologists are lacking Guidelines for patient referral and treatment to ensure uniformity of care not available Patient and family emotional, spiritual, information and communication needs not met due to deficiency of soft skills among HCW
Tapera et al. [18], 2020 Zimbabwe	To investigate palliative care knowledge and access among women with cervical cancer in Harare, Zimbabwe	A sequential explanatory mixed method study with descriptive cross-sectional surveys as a major study and qualitative inquiry as a minor study	134 women with cervical cancer 78 HCW involved in cervical cancer screening, treatment and palliative care 16 in-depth interviews 48 participants in 6 FGD 20 key informant interviews	Socially active people with responsibilities Advanced stage cancer Moderate level of education Unemployment rate is high No intimate partners Low palliative care Availability of PC skills among HCW Adequate training and guidance Poor PC referral Availability of medicine Limited PC knowledge among patients and HCW Limited access to PC Patient misconception about PC Misconception and/or limited knowledge of cervical cancer Misconception of PC Low affordability of medicines Poor implementation of PC policy	Mean age of patients 52 years SD ± 12 Cancers stage FIGO ≥ 2b2 94% Secondary education − 61% No income - 51%; Unemployed - 67% Widowed, divorced or separated 60% Received palliative care − 13% Mean age of HCW 37 years SD ± 12 Trained to provide PC – 72% HCW accessing PC guidelines – 76%; HCW referring patient to PC specialty unit – 1.2% Stock-outs of pain medicine – 22% Knowledge of palliative care among HCW and patients are limited Few patients access palliative care even among women who received other forms of treatment Patient perceives cervical cancer as a death sentence Cervical cancer diagnosis and palliative care is stigmatized and linked to near death Palliative care only provided by hospice High cost of medicines for cancer patients Palliative care policy framework available but not fully implemented
Kebede et al. [33], 2020- Ethiopia	To explore communication in cancer care in Ethiopia from the perspective of physicians, patients, and family caregivers	Ethnographic exploratory qualitative study using semi-structured interviews, and triangulating findings with direct observations and video-recordings of authentic interactions between physicians, patients, and family caregivers during hospital rounds	Purposively sampled participants 54 cancer patients • 20 males 34 females • Age 22–53 years • Cervical cancer number not mentioned 22 family caregivers • 11 male 11 female 16 physicians • 3 senior 13 junior • 11 males 5 females • Ages 29–58 years	Workload and time pressure Insufficient consultation time Insufficient information on disease Good HCW attitude Lack of privacy Language barrier No confidentiality No confidentiality Reluctance to reveal sensitive information Compromised autonomy Limited knowledge of cancer Long distances Influence from others Misconceptions about disease Culture/attitude Expectations Fear of stigmatization and isolation,	Large number of patients; few physicians Difficulty in allocating sufficient time for patient and family caregiver consultation Patient and family caregiver but not provided with enough information Care recipients described communication with physician positively; nice and humble doctors Consultation done in small non-sound-proof cubicles; frequent interruptions by other patients and staff Physicians and patients/family caregivers speaking different languages; Use of interpreters Family caregivers tended to dominate interaction with physicians including discussion on diagnosis and prognosis Male family member often responsible for making decisions and taking responsibility for patient care and expenses Patient has problems understanding the disease and treatment process Patient comes from distant rural areas Some patients told that traditional medicine is better; seeks professional help late after going through religious treatment with holy water Some care-recipients believe cancer is a curse from God; while others believe it can be transmitted from one person to another Stigma and taboo related to genitals in some communities; some female patient found it difficult to discuss cervical cancer openly and withheld vital information Hope to receive help from the doctor Patients reluctant to share diagnosis with others, including family
Tapera et al. [26], 2019 Zimbabwe	To investigate the determinants of access to cervical cancer treatment and palliative care services in Harare, Zimbabwe	Sequential explanatory mixed method	Phase 1 –quantitative survey: 148 healthy women 134 cervical cancer patients or survivors 78 health workers involved in cervical cancer Phase 2- 6 FGD with 8 members per group 16 in-depth interviews 20 key informant interviews	User’s location User’s attitude User’s resources User’s attitude or resources Self- efficacy/attitude Satisfaction with quality of care/Expectations Transportation / user’s resources /service location Demand for service / user’s resources / service location /user’s location Financial hardships Willingness to pay Cost and prices of service Over servicing/ characteristics of health services Financial hardship Misconceptions about disease and treatment Social factors / user’s attitude Acceptability	Compared to those who did not receive any care, most patients receiving treatment: • Lived in urban high density population areas, (p = 0.025) • Were of protestant faith, (p = 0.028), • With household heads being a professional (p = 0.038). Most patients receiving no treatment had household heads with no formal education (p = 0.038) Locus of control was positively associated with uptake of treatment Perception of competency of HCW positively associated with treatment uptake Walking as a means of reaching nearest health facility was negatively associated with perceptions of access Mostly lack of transport or high transport cost for rural people who travel long distances to reach few cancer centres Accommodation often required for patient and family at these centre paid for by family Patient buys own drugs High cost of diagnostic tests, medications and other treatment unaffordable to most Same test ordered multiple times at every level of care Loss of employment for patient and accompanying family member Cervical cancer seen as death sentence and radiotherapy introduces foreign material into the body. Influence of traditional and some religious healers, family, attitudes play a major role in seeking treatment Prolonged waiting period
Miranda et al. [27], 2016 Brazil	To analyse the clinical and socio- demographic profile of cancer patients seen in a specialized emergency service, considering the availability of palliative care and home care	Descriptive, cross-sectional study of medical records with an analytical component	191 medical reviews including those of 35 patients with cervical cancer	Characteristics of health services	Brazil has hierarchical and regionalized network that prevents 1 in 5 patients to be displaced from rural areas in the state for case review in a referral hospital
Maree & Langley, [7], 2014 South Africa	To elicit the experiences of underprivileged women being confronted with cervical cancer	Qualitative exploratory and contextual with descriptive and interpretive elements	19 purposively selected newly diagnosed cervical cancer patients being prepared to receive radiotherapy	Vulnerable population Anxiety and/or depression Misconceptions about cervical cancer Waiting for treatment / Characteristics of health services Dissatisfaction with health system Medical assistance available Financial hardship Out of pocket costs /Family support Unemployed / user’s resources HCW attitude Disclosing bad news Family support Cost of being away from work Alternative forms of treatment / attitudes Limited knowledge of disease Communication Limited knowledge of HCW Majority of patients has advanced stage cancer	Mean age 47.2 years (29–70) Majority (16/19) were black South Africans. Most patients experience worrying symptoms such as excessive vaginal bleeding, offensive discharge, and pain Some patients experienced bleeding but did not have pain so they never though they had serious illness It took an average of 17 months from first symptoms to the start of specific treatment Patients felt the public health system failed them in terms of prompt diagnosis and start of treatment Patients do not pay for treatment in the public health system Private health system available and faster but patient could not afford the cost of treatment here unless paid for by relatives Most patients were unemployed The doctors were nice to the patients but had difficulty communicating bad news Some patients preferred to be alone when bad news was broken Found it difficult to disclose their diagnosis to the family to protect them from distress Patients who received their diagnosis in presence of family felt better after being consoled Accompanying family stayed away from work for consultations Seeking conventional medicine was delayed as some patients were advised by relatives to attend spiritual and traditional healing One patient did not know that cervical cancer is life threatening Most patients wanted to know more about the disease and treatment but the doctors could not explain to them Cancer stage 1b – 2a : 3 patients Cancer stage 2b to 4 : 15
Dutta et al. [28], 2013 India	To find out the socio-demographic causes which lead to non-compliance to treatment even after registration for radiotherapy	Retrospective analysis of medical records	144 patients with cancer of the cervix already registered to receive treatment They received initial treatment at a nearby centre but had to go for the second phase of treatment at a centre 567 km away. Transport vouchers were provided but patients had to find and fund their own accommodation	Family burden Dependency / resources Older age Low literacy level Transport difficulties and high cost Burden of family responsibility Social status	Majority of patients were postmenopausal (56.94%), and had family burden of > 3 children Only 6.25% were self-employed The rest depended on family and husband for their livelihood Elderly patients > 50 were most unlikely to complete their course of treatment High number of illiterate women were unlikely to complete their treatment Most patient living more than 100 km from the nearest treatment facility unlikely to complete their course of treatment Women with more children preferred to take care of their families rather than be away from home for prolonged period of time Women who completed their treatment in time were wealthier; middle-aged; with < 3 children
Mwaka et al. [31], 2013 Uganda	To explore the perceptions of operational level healthcare professionals who work directly with cervical cancer patients on challenges faced by women seeking cervical screening, cervical cancer diagnosis and management, and challenges faced by health professionals in providing cervical cancer care	Qualitative inquiry using key informant interviews	10 female nurse/midwives, 2 gynaecologists, 2 medical officers and 1 surgeon working in a public regional and a mission hospital in northern Uganda	Patients and community related factors Healthcare professionals deficiencies Health facility related factors Facilities Health policy factors	Lack of awareness on cervical cancer and available services Discomfort with exposure of women’s genitals Perceived pain during pelvic examinations Men’s lack of emotional support to women Inadequate knowledge and skills about cervical cancer management Long distance to care centres Few gynaecologists and lack of pathologists Delayed histology results Lack of morphine for pain control Lack of specialized cancer treatment facilities Lack of vaccination policy for HPV Large number of women presenting with late stage cervical cancer
Van Schalkwyk et al. [32], 2008 South Africa	To gain an understanding of the routes that women presenting with advanced cervical cancer followed, from experiencing the first signs and symptoms of disease until they received radiotherapy	Exploratory qualitative phenomenological study of the subjective experiences of women with cervical cancer	15 consecutive women with advanced (stage 2b and worse) cancer of cervix	Limited knowledge Anxiety, fear of the unknown Stigmatization Self-efficacy Long waiting period for treatment, even longer for rural women Limited knowledge and awareness of HCW (Low index of suspicion) Culture of secrecy, taboo regarding reproductive organs Influence of significant others Misconceptions and limited knowledge of the disease Positive support from family Negative attitude of family and misinformation Dissatisfaction	At first symptoms (bleeding, pains, discharge) most women knew something was wrong but lacked the knowledge of what was required to manage the problem Most experienced concern, sadness, embarrassment, sadness and isolation People avoided them in public spaces due to the bad smell Were able to seek treatment once they knew what the problem was Average time from diagnosis to treatment was 17.3 months (11.8 for urban vs. 28.4 for rural women) First contact with HCW did not result in correct diagnosis at any level of care Some women did not report their symptoms due to embarrassment but instead only complained of minor problems Traditional healers were consulted as a result of advice or insistence of support persons Some believed that their problem was caused by demons and attended traditional healers first, going to hospital only when they did not get relief Some family members, intimate partners, workplaces, and the church were very understanding and supportive Others accused the patient of immorality, and did not allow them in church because of being unclean Most patients were not happy with the way they were treated in the healthcare facilities
Noor-Mahomed et al. [30], 2003, South Africa	To identify suicidal risk-factors, psychological morbidity, coping, and role of social support in cervical cancer patients	Prospective cross-sectional	21 in-patients of which 13 were planned for palliative, and 8 for radical radiotherapy		Low level of satisfaction with social support Fewer of no visit from husbands and boyfriends Inadequate social support from significant others Feeling of being a burden to others Stigmatised by society Relatives lived far from the hospital

Of the 12 studies there were 2 retrospective cross-sectional [27, 28], 3 prospective cross-sectional [4, 29, 30], 5 qualitative [7, 25, 31–33], and 2 mixed-method designs [18, 26]. There were no randomised control trials. Ten studies were conducted in Africa [4, 7, 18, 25, 26, 31–33] and one each from Brazil [27] and India [28]. Within the 12 studies, 911 unique women with cervical cancer, 148 healthy women, 424 family members, 78 healthcare workers involved with cervical cancer, and 20 informal caregivers were included. Findings from all the studies were collated under 10 sub-constructs alongside four main constructs of access to healthcare [24] [Table 3].

**Table 3:** Access domains and sub-domains

Domains	Geographic accessibility		Availability				Financial accessibility			Acceptability		
Subdomains Articles	Service location	User’s location	Health workers	Drugs	Equipment	Demand for services	Cost & prices of service	User’s resources	User’s willingness to pay	User’s attitude	User’s expectations	Characteristics of health services
Bates et al., [4]	•	•				•		•		•		•
Dutta et al., [28]	•	•						•				
Kabebew et al., [29]			•			•					•	•
Kebede et al.,[33]	•	•	•			•				•	•	•
Maree et al., [7]			•			•			•	•	•	•
Miranda et al., [27]	•	•				•						•
Mwaka et al.,[31]	•	•	•	•	•	•	•	•		•		•
Noor-Mahomed et al., [30]	•	•								•	•	
Tapera et al., [25]	•	•	•		•	•	•	•				•
Tapera et al., [18]			•	•		•		•	•	•		•
Tapera et al., [26]	•	•		•		•	•	•		•		•
Van Schalkwyk et al., [32]	•	•	•			•				•	•	•

Below, we present the findings of thematic analysis of the review under the following domains: geographic accessibility, availability, financial accessibility, and acceptability.

### Geographic accessibility

Health facilities were found in central referral hospitals located in urban centres far from the rural areas where a majority of patients lived [4, 18, 27–33]. Most patients travelled long distances, usually by public transport, or by walking to the nearest facility to access cancer treatment or primary care for pain and symptom relief [34].

### Availability

The demand for palliative care services and unmet needs featured prominently in all studies except one [33]. Three studies [18, 31, 26] reported lack or shortage of essential medicines used in palliative care such as morphine for pain control. One study [31] reported shortage of blood for transfusion of cervical cancer patients presenting in shock after excessive bleeding. Unavailability of radiotherapy equipment is reported in 2 studies [25, 31] with each of the countries of origin possessing only one old-type radiotherapy equipment (cobalt-60) for the entire population. Seven studies reported on the availability of healthcare workers. Shortage of specialists in oncology, gynaecology, pathology, and PC was reported in 2 studies [25, 31] while limited knowledge of cervical cancer management and PC skills among healthcare workers was reported in 6 studies [7, 18, 26, 31–32].

### Financial accessibility

High cost of radiotherapy and pathology services, unaffordable by a majority of patients and families was reported in three studies [18, 26, 31]. Only 1 study from South Africa found that radiological, radiotherapy, laboratory and consultation services were covered by the state in the public sector, but was still expensive in the private sector [7]. In most studies, patients and their family were poor with limited financial resources [4, 7, 18, 26, 31, 33]. Financial hardship was reported in nearly all studies. In Malawi, most households, especially those from the rural areas lived in extreme poverty at ≤$1.89 per day [4]. Families were willing to pay for healthcare, mainly through out-of-pocket expenditure [18, 28, 31, 32]. Some households experienced catastrophic cost as a result, to the extent of dissaving or selling property to cover healthcare costs [4]. In a few studies, basic healthcare costs were covered or subsidised by government [4, 7, 28, 32], while in some family members shared costs with the government while non-governmental organizations provided support for subsistence needs [4, 31]. Substantial component of costs incurred by patients and families went towards covering the costs for travel, subsistence and accommodation while seeking or attending treatment in distant centres [18, 28, 31].

### Acceptability

Characteristics of health services were preeminent in all the studies analysed of which most of them revealed scarcity of service points with significant physical distance away from users and associated difficulties accessing the needed services. In addition, the existing facilities were ill- equipped with frequent radiotherapy interruptions due to breakdown and stock-outs of essential pain medicine such as morphine [18, 31]. Overcrowding in the consultation rooms with lack of privacy [33], healthcare staff with insufficient knowledge and skills to provide disease information that meets the need of the patient and family [7, 18, 31, 32], and in some instance over-servicing with multiple radiological and other tests ordered at numerous service delivery points [18] were some of the challenges reported. Communicating unfavourable information to patients about their illness and addressing emotional needs was also poorly handled by staff in some studies [7, 25, 26].

Misconceptions about the nature of cervical cancer, its treatment, and embarrassment on exposure of their body together with the culture of privacy associated with female genitals also prevented the patients from receiving prompt care [26–28]. Patient or caregiver satisfaction with healthcare providers and available services was highlighted in a few studies [7, 26, 28]. However, other studies revealed dissatisfaction mainly due to long waiting period before treatment [26, 29, 32] and high cost of services [26, 27, 31].

User’s attitudes reported in the studies included cultural beliefs and attitude towards healthcare services visited. Some patients preferred traditional medicine or religious healing to biomedical care due to pressure from significant people in their lives [26, 28, 31] or misconceptions about the cause of their illness [28]. Other patients stayed away from conventional services due to stigma their communities associated with cancer or its symptoms such as offensive vaginal discharge and bleeding, including misconceptions that cancer can be transmitted directly on contact with a patient, or that the patient was also carrying Human immunodeficiency virus (HIV) and suffering from Acquired Immune Deficiency Syndrome [AIDS] [30, 32, 33].

Family responsibilities were also reported to affect access to health care. In one study, widowed, divorced, or separated cervical cancer patients who were the only income earners for their family through formal or informal self-employment could not afford prolonged absence from their jobs while seeking or receiving treatment [18]. Family burden was also a factor in women with more than 3 children who preferred to stay at home to care for their families even though travel vouchers to treatment centres were provided [28, 33]. Another study reports that women’s responsibility of cultivating their farms preceded their need for healthcare and besides they first had to get permission from their husbands to be able to seek treatment [31]. In some households, patients chose to forgo treatment rather than cause financial difficulties to their families [4]. Yet other patients requested health care staff not to reveal their diagnosis for fear of causing anxiety to their family members [7, 33].

## Discussion

This systematic review reveals numerous palliative care access challenges, perceived and experienced, by women with advanced cancer of the uterine cervix and/or their caregivers in the LMICs. The challenges are both personal and system-based, emanating from all the four domains of access to quality healthcare such as lack of physical accessibility, unavailability of quality services, poverty and poor affordability, and barriers to acceptance and use of available services by those in need.

Despite significant progress made in the development of palliative care in Africa [35, 36], services are still not universally integrated into the healthcare system of many countries and remain inconsistent, occurring in isolated centres with limited geographical access by a majority of the population in need [37]. However, limited access as a consequence of geographic location is not unique to the developing world as found in this systematic review. In developed countries such as the United States of America (USA) many patients still lack access to PC because of unfavourable geographic location or distance from appropriate treatment facilities [38]. A study conducted in the USA among patients with gynaecological cancer found that patients with advanced ovarian or cervical cancer residing more than 45 miles from the healthcare provider were less likely to utilize PC compared to those living within less than 2 miles [39]. Another study among cervical cancer patients investigating how distance to healthcare facility influence initiation of treatment revealed that patients residing 15 miles from the nearest treatment facility were less likely to initiate timely treatment compared to those less than 5 miles [40]. Geographic accessibility involves the physical distance or travel time from the user’s location to the service delivery location. Previous studies support the significant role of the geographic accessibility by demonstrating an inverse relationship between distance and travel time to service delivery points and the use of healthcare services [41, 42]. Good roads and adequate communication enhances geographic accessibility [24].

The demand for PC services and unmet needs featured prominently in all studies analysed in this review. Accessing the right type of care that meets the demand of those who need it is central to availability [24]. Availability concerns the opportunity to access the right type of healthcare promptly whenever needed. It involves the availability of knowledgeable and skilled healthcare providers, medicines, equipment, acceptable opening hours, and acceptable waiting times [24]. Report from literature reiterate the findings of this study that most cervical cancer patients and their families live in the LMICs where availability of quality palliative care is minimal despite the high demand [43]. Internationally, the American Society of Clinical Oncology (ASCO) recommends that a coordinated system to assess and meet PC needs of patients and family should be made available at all levels of a healthcare system [44].

Available evidence from diverse resource settings suggest that women with cancer of the cervix frequently suffer from severe complex refractory symptoms not readily relieved by basic PC [45, 46]. Addressing these symptoms require more advanced procedures such as palliative external beam radiotherapy to control vaginal bleeding and discharge, advanced medical therapies, nerve block for intractable neuropathic pelvic pain, surgical procedures for bowel obstruction, and psycho-oncology to manage severe or refractory anxiety and depression [45]. Identifying needs of patients and their families, communication, assessment and treatment of pain and other symptoms, and referral for management of complex distressing symptoms is recognised as a basic requirement even at lower levels of care [44, 47]. Many African countries still lack the ability to provide quality PC due to inadequate infrastructure, staff shortages, low doctor-patient ratio and paucity of trained PC specialists [48] compounded by insufficient supply of morphine, an opioid considered by World Health Organisation as an essential medicine in treatment of pain, including cancer pain [49].

Poverty, loss of income and inability to afford basic subsistence and healthcare-related costs was a recurring observation in the review. Financial accessibility includes cost and prices of services, cost of time, users’ resources, and users’ willingness to pay for the services [24]. Distance to health facilities has a significant bearing on the affordability especially for rural dwelling population incurring travelling, food and accommodation costs in addition to the direct cost of treatment. Unlike in the developed countries where medical insurance covers healthcare expenses [50–52], there is low medical insurance coverage in most LMICS and cost of treatment for people with serious illness is borne by patients and family mainly as out-of-pocket expenses [42, 49, 53]. For instance, in South Africa only 16.4% of the population is covered by a medical aid [54]. In an instance of extreme poverty, a proposal has been made for accessible social support for any patient in need of PC and for their main caregiver to include transportation vouchers, cash payments, food packages, and other types of in-kind support [53].

Acceptability of heath care services involves factors such as characteristics of health services, and user’s attitudes and expectations [24]. It encompasses how satisfied users are with the services provided. It also depends on care receiver’s attitude and cultural beliefs. Factors such as opening hours, geographic distance, availability of medicines, staff complement, attitude and knowledge, and costs, determine acceptability of an available PC service for patients and their family. In this review, patients and family were not satisfied with the services as s a result of difficulties they encountered. The difficulties varied from long distance of travel to perception of poor communication with the staff. However, other factors were related to socio-cultural beliefs and influence from significant others [32].

Lack of knowledge and awareness of available services, distrust of the healthcare system, traditional gender roles and language barriers have been reported elsewhere as factors that limit utilization of health services [55]. Lack of knowledge has been reported to hinder the use of PC services in developed countries also [56]. Community belief in the superiority and effectiveness of traditional medicine in treatment of cervical cancer and beliefs in spiritual healing also hinder utilization of biomedical health services in some LMICs [30, 33, 57, 58].

## Strengths

To the researchers’ knowledge this is the first systematic review that has looked into access to PC in women with advanced cervical cancer and their families in the LMICs. The use of thematic synthesis has the potential to draw conclusion based on common elements from studies of diverse designs [23]. This may allow for generating hypothesis that may form a structure for future research in this field to investigate culturally sensitive and acceptable PC provision.

## Limitations

As only studies published in English were considered in the inclusion criteria, it is possible that there are studies published in other languages that could have been included also. Other limitations could be that the included studies used different study designs (qualitative, quantitative, mixed methods) and different outcomes or no validated outcome measures as such could not allow for meta-analysis. Additionally the relatively small number of studies included in the review may have limited the reliability of conclusions that could be drawn.

## Implication for practice

Understanding the lived experiences and factors that primarily concern cervical cancer patients and their caregivers with respect to accessing PC services creates a better understanding of barriers, facilitators, and measurable factors that can act as basis of remedial actions to improve access and enable the development and implementation of PC models that specifically address needs of those affected. Establishing adequately staffed and equipped facilities within reach of the majority of the population served will help bring services close to the people.

## Conclusion

Findings of this systematic review suggest that palliative care for cervical cancer patients is still not universally available especially in the LMICs. Cervical cancer patients often suffer significant distressing symptoms which could be alleviated by the provision of effective palliative care. As the development of the practice of palliative care gradually improves in the developing countries, concerted effort must be made to enhance access to cervical cancer patients and their families so as to improve their quality of life and disease experience.

### Electronic supplementary material

Below is the link to the electronic supplementary material.

Supplementary Material 1

## Data Availability

The authors confirm that the data supporting the findings of this study are available within the article and/or its related files. Any other information that support the findings of this study are available from the corresponding author, [F.O], upon reasonable request.
